# Indoleamine 2,3-Dioxygenase Activity During Acute Toxoplasmosis and the Suppressed T Cell Proliferation in Mice

**DOI:** 10.3389/fcimb.2019.00184

**Published:** 2019-06-05

**Authors:** Christoph-Martin Ufermann, Andreas Domröse, Timo Babel, Anne Tersteegen, Sevgi Can Cengiz, Silvia Kathrin Eller, Katrin Spekker-Bosker, Ursula Regina Sorg, Irmgard Förster, Walter Däubener

**Affiliations:** ^1^Institute of Medical Microbiology and Hospital Hygiene, Heinrich-Heine-University Düsseldorf, Düsseldorf, Germany; ^2^Immunology and Environment, Life and Medical Sciences (LIMES) Institute, University of Bonn, Bonn, Germany

**Keywords:** *Toxoplasma gondii*, IDO, T cell suppression, mouse, kynurenine

## Abstract

*Toxoplasma gondii* (*T. gondii*) is an obligate intracellular parasite and belongs to the phylum Apicomplexa. *T. gondii* is of medical and veterinary importance, because *T. gondii* causes the parasitic disease toxoplasmosis. In human cells, the interferon-gamma inducible indoleamine 2,3-dioxygenase 1 (IDO1) is an antimicrobial effector mechanism that degrades tryptophan to kynurenine and thus limits pathogen proliferation *in vitro*. Furthermore, IDO is described to have immunosuppressive properties, e.g., regulatory T cell differentiation and T cell suppression in humans and mice. However, there is only little known about the role of IDO1 in mice during acute toxoplasmosis. To shed further light on the role of mIDO1 *in vivo*, we have used a specifically adjusted experimental model. Therein, we infected mIDO1-deficient (IDO^−/−^) C57BL/6 mice and appropriate wild-type (WT) control mice with a high dose of *T. gondii* ME49 tachyozoites (type II strain) *via* the intraperitoneal route and compared the phenotype of IDO^−/−^ and WT mice during acute toxoplasmosis. During murine *T. gondii* infection, we found mIDO1 mRNA and mIDO1 protein, as well as mIDO1-mediated tryptophan degradation in lungs of WT mice. IDO^−/−^ mice show no tryptophan degradation in the lung during infection. Even though *T. gondii* is tryptophan auxotroph and rapidly replicates during acute infection, the parasite load was similar in IDO^−/−^ mice compared to WT mice 7 days post-infection. IDO1 is described to have immunosuppressive properties, and since T cell suppression is observed during acute toxoplasmosis, we analyzed the possible involvement of mIDO1. Here, we did not find differences in the intensity of *ex vivo* mitogen stimulated T cell proliferation between WT and IDO^−/−^ mice. Concomitant nitric oxide synthase inhibition and interleukin-2 supplementation increased the T cell proliferation from both genotypes drastically, but not completely. In sum, we analyzed the involvement of mIDO1 during acute murine toxoplasmosis in our specifically adjusted experimental model and found a definite mIDO1 induction. Nevertheless, mIDO1 seems to be functional redundant as an antiparasitic defense mechanism during acute toxoplasmosis in mice. Furthermore, we suggest that the systemic T cell suppression observed during acute toxoplasmosis is influenced by nitric oxide activity and IL-2 deprivation.

## Introduction

The apicomplexan parasite *Toxoplasma gondii* (*T. gondii*) is, due to the fact that it can infect nearly all warm-blooded animals, considered to be the most successful parasite worldwide. A primary *T. gondii* infection in humans is usually asymptomatic, but can cause congenital toxoplasmosis and can thus lead to fatal consequences for the fetus or newborn. In immunocompetent individuals, *T. gondii* establishes a chronic infection and will thus persist lifelong in the host. Reactivation of a chronic *T. gondii* infection in humans—e.g., during immunosuppression—results in cerebral toxoplasmosis in most cases (Schlüter et al., 2014). A recent study by Wilking et al. (2016) showed that *T. gondii* infection, while depending on demographic factors, is highly prevalent in Germany; about 55% of the representative cohort was seropositive for *T. gondii*.

Defense mechanisms directed against *T. gondii* are intensively studied. In addition, extensive data were obtained analyzing murine toxoplasmosis since the mouse model is the preferred animal model to study toxoplasmosis *in vivo* (Gazzinelli et al., 2014; Yarovinsky, 2014; Sasai et al., 2018).

Many different effector mechanisms are described to be involved in the defense against *T. gondii*, including iron depletion (Dimier and Bout, 1998) and enhanced autophagy (Krishnamurthy et al., 2017). However, the most frequently studied mechanisms directed against intracellular parasites in mice are the enhanced production of nitric oxide (NO) by inducible nitric oxide synthase (iNOS) (Adams et al., 1990; Khan et al., 1997) and the activity of GTPases, enzymes that can hydrolyze guanosine triphosphate (Hunn et al., 2011; Degrandi et al., 2013; Sasai et al., 2018). In humans, tryptophan depletion by indoleamine 2,3-dioxygenase (IDO) is the most frequently described defense mechanism (Pfefferkorn, 1984; MacKenzie et al., 2007).

iNOS was found to be effective against *T. gondii* in cell cultures, e.g., murine macrophages (Adams et al., 1990) or murine mesenchymal stem cells (Meisel et al., 2011), and more importantly, in *in vivo* studies using iNOS-deficient mice (Khan et al., 1997). However, in contrast to these findings, NO production favored the growth of *T. gondii* in cytokine-activated human uroepithelial cells (Däubener et al., 1999), human hepatocytes (Bando et al., 2018), and human retinal pigment epithelial cells (Spekker-Bosker et al., 2019).

Another important antiparasitic effector mechanism directed against *T. gondii* is the induction of GTPases such as immunity-related GTPases (IRGs) (Hunn et al., 2011) or murine guanylate binding proteins (mGBPs) (Degrandi et al., 2013; Sasai et al., 2018). In humans, there is only one IRG present, and this human IRG is not interferon inducible (Bekpen et al., 2005). Furthermore, human GBP-mediated antiparasitic mechanisms differ from those of murine GBPs (Hunn et al., 2011; Johnston et al., 2016).

The role of the interferon-gamma (IFN-γ) inducible IDO in the defense against *T. gondii* was first recognized *in vitro* using human fibroblasts (Pfefferkorn, 1984) and has been confirmed in other human cell lines (e.g., epithelial and endothelial cells) (MacKenzie et al., 2007). In contrast, in murine cells, mIDO does not mediate defense against intracellular *T. gondii* tachyzoites after IFN-γ stimulation as shown in macrophages and mesenchymal stroma cells (Schwartzman et al., 1990; Meisel et al., 2011). Interestingly, another isoform of IDO has been reported, named indoleamine 2,3-dioxygenase 2 (IDO2). IDO2 has a lower tryptophan affinity than IDO1 *in vitro*, and its induction, expression, as well as distribution are described to be different from IDO1 (Yeung et al., 2015).

Despite the abovementioned differences in the IDO-mediated antiparasitic effects between human and murine cells, IDO-mediated immunoregulatory effects have been described in both murine and human cells. For example, the group of Munn and coworkers found an important function for IDO in the development of immune tolerance in allogeneic pregnancy in mice and described a profound immunosuppression mediated by IDO-positive human macrophages (Munn et al., 1998, 1999). Furthermore, they described the tryptophan depletion as a possible reason for the inhibited T cell proliferation (Munn et al., 1998, 1999); thus, we suggest that this local reduction of tryptophan could also cause a local antimicrobial environment. The immunosuppressive activity of IDO has been confirmed by several groups and is of interest in transplant medicine as well as in tumor immunology and autoimmunity as reviewed previously (Löb et al., 2009). In sum, it was found that IDO-positive dendritic cells (DCs) are able to induce tolerance during T cell activation, while within the tissue, IDO-positive non-professional antigen-presenting cells such as fibroblasts and endothelial cells could inhibit the effector function of T cells (Lee et al., 2017).

Since the aforementioned effector mechanisms directed against *T. gondii* are differentially regulated in different species as well as cell types, we decided to investigate the influence of IDO on acute toxoplasmosis in a specifically adjusted murine experimental *in vivo* model.

## Materials and Methods

### Cell Line and Parasite Strain Cultivation

Human foreskin fibroblasts (HFF; ATCC® SCRC-1041™, Wesel, Germany) and the murine macrophage cell line (RAW 264.7; ATCC® TIB-71™, Wesel, Germany) were cultured in Iscove's modified Dulbecco's medium (IMDM; Life Technologies, Carlsbad, USA), supplemented with 5% (vol/vol) heat-inactivated fetal bovine serum (FBS; BioWhittaker®, Lot N°: 9SB003, Lonza, Basel, Switzerland). Cells, as well as isolated cells for *ex vivo* cultivation, were kept in a humidified Heraeus BB 6220 CO_2_ incubator (Thermo Fisher Scientific, Waltham, USA) (37°C, 5% CO_2_). HFF cells were passaged after confluency was reached using 0.05% trypsin/ethylenediaminetetraacetic acid (EDTA), (Life Technologies, Carlsbad, USA). Confluent HFF monolayers were used as host cells.

*T. gondii* strain ME49 tachyzoites (ATCC® 50611, Wesel, Germany) were maintained *in vitro* by serial passages in HFF. For infection experiments, parasites were propagated in HFF (for 42–48 h). Parasites were harvested by scraping off parasitized HFFs in phosphate-buffered saline (PBS) (Life Technologies, Carlsbad, USA). Intracellular parasites were syringe-released and dissociated from host cells debris by differential centrifugation [85 × *g*, room temperature (RT), 5 min; 780 × *g*, RT, 5 min]. Parasites were resuspended in PBS, counted, and adjusted to 5 × 10^5^ tachyzoites/ml.

### Animals and Infection Experiments

mIDO1-deficient (IDO^−/−^) mice (B6.129-*Ido1*^*tm*1*Alm*^/J) were originally obtained from the Jackson Laboratory (Bar Harbor, Maine, USA) and had a C57BL/6 genetic background. IDO^−/−^ mice were bred and kept under specific pathogen-free (SPF) conditions in the Central Unit for Animal Research and Animal Welfare Affairs of the Heinrich-Heine-University Düsseldorf. C57BL/6 (C57BL/6JRj) mice purchased from Janvier Labs (Le Genest-Saint-Isle, France) were used as wild-type (WT) controls. All experiments were performed with age- and sex-matched cohorts. Mice were infected intraperitoneally (i.p.) with 10^5^ *T. gondii* ME49 tachyzoites in 200 μl of PBS. Naïve control mice and infected mice were kept under SPF conditions and were checked daily. For sample collection, mice were euthanized by cervical dislocation 7 days post-infection (dpi). This study was performed in strict compliance with the German Animal Welfare Act. The experiments were authorized by the North Rhine-Westphalia State Agency for Nature, Environment and Consumer Protection (Permit# 84-02.04.2013.A271, 84-02.04.2013.A495, and 84-02.04.2016.A508). All efforts were made to minimize animal suffering during the experiments.

### Sample Collection

Blood samples were taken by cardiac puncture, and the sera were generated from clotted blood samples (4°C overnight) in two centrifugation steps (20,000 × *g*, 4°C, 10 min). Organs [lung, brain, liver, spleen, and mesenteric lymph nodes (MLNs)] were collected and washed in PBS. Whole lung, brain, and liver were homogenized in PBS using the Percellys® lysing kit CK28 and the Percellys® Minilys® tissue homogenizer (Bertin Instruments, Montigny-le-Bretonneux, France). All samples were stored at −80°C for further processing.

### Western Blot Analyses

The protein contents of supernatants from centrifuged tissue homogenates or cell lysates generated by freeze–thaw were determined *via* the Bradford assay (Bio-Rad Laboratories, Hercules, USA). Electrophoretic separation of proteins (30 μg protein per lane) was done with 10% NuPAGE Novex Bis-Tris Mini gels in the appropriate electrophoresis system (Thermo Fisher Scientific, Waltham, USA). Proteins were semi-dry blotted on nitrocellulose membranes (CarboGlas, Schleicher & Schüll, Dassel, Germany). Membranes were blocked in 5% (w/v) skim milk powder in PBS for 1 h at RT. For specific protein detection, the primary antibodies for murine β-actin (1:10,000) (AC-15, Sigma-Aldrich, Munich, Germany), murine iNOS (1:1,000) (1131-1144, CalBiochem®, Munich, Germany), or murine IDO (1:500) (AB9900, Chemicon, Merck Millipore, Billerica, MA, USA) were diluted in 0.5% (w/v) skim milk powder in PBS. Membranes were incubated for 1.5 h at RT and were washed three times with PBS (5 min each). The peroxidase-conjugated, secondary antibodies goat anti-mouse IgG (for mβ-actin) or goat anti-rabbit IgG (for mIDO and miNOS) (1:10,000–70,000, Jackson ImmunoResearch Laboratories, Dianova, Hamburg, Germany) were diluted in 0.5% (w/v) skim milk powder in PBS. Membranes were incubated for 2 h at RT and were washed three times with PBS (5 min each). Labeled proteins were detected by enhanced chemiluminescence (Amersham Pharmacia Biotech, Freiburg, Germany).

### qRT-PCR Analysis of Transcript Levels

Total RNA was extracted according to the TRI Reagent protocol (Merck, Darmstadt, Germany). Briefly, total RNA was extracted from 50 μl of lung tissue homogenate with 500 μl of TRI Reagent and 100 μl of chloroform followed by precipitation with isopropyl alcohol. Extracted RNA was dissolved in 40 μl of UltraPure™ distilled water (Thermo Fisher Scientific, Waltham, USA) and RNA concentration was determined *via* NanoDrop (Thermo Fisher Scientific, Waltham, USA). Reverse transcription of 1.5 μg of total RNA to cDNA was performed with M-MLV reverse transcriptase and oligo(dT) 12–18 primers according to the manufacturer's instruction (Thermo Fisher Scientific, Waltham, USA). PCR primers to amplify the genes of interest were designed using the Universal ProbeLibrary Assay Design Center (Roche, Basel, Switzerland) and are listed in Supplementary Table S1. Real-time PCR was performed with the Takyon NoRox Probe MasterMix dTTP (Eurogentec, Lüttich, Belgium) on a BioRad CFX96 Touch Real-Time PCR Detection System (Bio-Rad Laboratories, Hercules, USA). Quality of qPCR analysis was verified by technical replicates for each sample in each run. Each well of a multiplate 96-well PCR plate contained 5 μl of cDNA template, 12.5 μl of Takyon NoRox Probe Master Mix dTTP, 0.3 μl of primer (10 μM each), 0.5 μl of probe (10 μM), and 6.4 μl of H_2_O for a total reaction volume of 25 μl. The PCR conditions were 7 min at 95°C and 40 cycles of 95°C for 20 s and 60°C for 1 min.

### Tryptophan and Kynurenine Quantification

We used high-performance liquid chromatography (HPLC) analysis to quantify total free tryptophan and kynurenine in mice serum and lung tissue. To precipitate existing proteins within the samples, they were mixed with trichloroacetic acid (2.5% final concentration; Sigma-Aldrich, Munich, Germany). To monitor measurement quality, all samples were mixed with 3-nitro-L-tyrosine (Sigma-Aldrich, Munich, Germany) with final concentrations of 2.5 or 10 μg/ml for lung tissues or sera, respectively, as internal standard. All samples were filtered (pore size 0.22 μm) prior to injection.

Analysis was performed with a System Gold® HPLC system (Beckman Coulter, Krefeld, Germany) under usage of a module 166 UV/VIS detector. For separation, a reverse-phase C18 column cartridge (Purospher® STAR RP-18 endcapped, Sorbent Lot No. FC095368, 3-μm particle size, 55-mm length, 2-mm diameter; Merck, Darmstadt, Germany) with an adequate guard column (Purospher® STAR RP-18 endcapped, Sorbent Lot No. HX435803, 5 μm particle size, 4 mm length, 4 mm diameter; Merck, Darmstadt, Germany) was used in a manuCART® 55-mm cartridge holder (Merck, Darmstadt, Germany). The mobile phase consisted of 50 mM sodium acetate (Merck, Darmstadt, Germany) adjusted to pH 4.2 with acetic acid (Merck, Darmstadt, Germany) with 5 or 2% acetonitrile (Merck, Darmstadt, Germany) for tryptophan and kynurenine analysis, respectively, using a flow of 0.5 ml/min. All eluents were purchased at least as gradient grade and underwent a vacuum degassing as well as a filtration with a 2 μm filter. The absorbance was measured at 280 nm for tryptophan and 360 nm for kynurenine; calculation occurred on the basis of previously measured calibration curves with purchased highly pure L-tryptophan and L-kynurenine (Sigma-Aldrich, Munich, Germany).

### qPCR Analysis of the Parasite Load

DNA was extracted from lung tissue homogenate by proteinase K digestion. In brief, 500 μl of digestion buffer (1% proteinase K (200 μg/mL; Qiagen, Venlo, Netherlands) in lysis buffer [100 mM Tris/HCl (pH 8.5), 5 mM EDTA (pH 8), 0.2% SDS, and 200 mM NaCl] was added to 20 μl of lung tissue homogenate and was incubated at 56°C and 1,100 rpm on a thermo-shaker for 90 min. DNA was precipitated with 500 μl of isopropyl alcohol and washed with 500 μl of 70% ethanol. Extracted DNA was dissolved in 50 μl of UltraPure™ distilled water and the DNA concentration was adjusted to 100 ng/μl. Quantitative real-time PCR (qPCR) was performed with the abovementioned detection system. For parasite quantification, a standard curve with adjusted *T. gondii* genomic DNA concentrations was established. The oligonucleotides and template-specific probe that were used are listed in Supplementary Table S1. These oligonucleotides bind to a sequence segment of the 35-fold repetitive B1 gene of *T. gondii* that is commonly used in diagnostics (Burg et al., 1989; Pelloux et al., 1998). Quality of qPCR analysis was verified by technical replicates for each sample in each run. Each well of a multiplate 96-well PCR plate contained 5 μl of DNA template, 12.5 μl of Takyon NoRox Probe Master Mix dTTP, 2.5 μl of primer (3 μM each), and 2.5 μl of probe (2 μM) for a total reaction volume of 25 μl. The PCR conditions were 10 min at 95°C and 45 cycles of 95°C for 15 s and 60°C for 1 min.

### Isolation and Cultivation of Murine Cells From Spleen and Mesenteric Lymph Nodes

For *ex vivo* lymphocyte proliferation experiments, cells from spleen and MLN tissues were digested using 1 mg/ml collagenase (C2139, Sigma-Aldrich, Munich, Germany) and 180 U/ml DNase (Roche, Basel, Switzerland) in PBS for 30 min at 37°C. Digested tissues were passed through 70-μm nylon sieves (Falcon® Corning Inc.; Corning, New York, USA) followed by erythrocyte lyses (MORPHISTO GmbH; Frankfurt am Main, Germany). Cells were resuspended in medium [IMDM with 5% FBS and 100 U/ml penicillin/100 μg/ml streptomycin (Biochrom GmbH, Berlin, Germany)] and counted using trypan blue (0.4%; Sigma-Aldrich, Munich, Germany). Cells were seeded in low-evaporation lid 96-well flat-bottom plates (Corning Inc., Corning, New York, USA) at 3 × 10^5^ cells per well.

### Lymphocyte Proliferation Assay

Lymphocyte proliferation was stimulated with the mitogens concanavalin A (ConA; 1 μg/ml; Sigma-Aldrich, Munich, Germany) and the class B phosphate-linked cytosine and guanine oligonucleotide ODN1826 (CpG B; 0.1 μM; Invivogen; San Diego, CA, USA) as indicated. Additional supplementation with recombinant human interleukin-2 (IL-2; 5 ng/mL; R&D Systems, Minnesota, USA) and the NOS inhibitor N^G^-monomethyl-L-arginine (N^G^MMA; 100 μg/ml; Merck, Darmstadt, Germany) was performed in the concentrations indicated.

Lymphocyte proliferation was determined by the ^3^H-thymidine incorporation method. In brief, ^3^H-thymidine (74 kBq per well; GE Healthcare Buchler GmbH & Co. KG, Braunschweig, Germany) was added 48 h post-stimulation. Lymphocyte proliferation was stopped after additional 24 h of cultivation by freezing. Lymphocyte proliferation was determined by measuring incorporated ^3^H-thymidine using liquid scintillation spectrometry (1205 Betaplate, PerkinElmer, Jugesheim, Germany).

### Indirect Nitric Oxide Estimation

NO production was measured *via* the Griess assay (Ding et al., 1988). Here, nitrite—a stable breakdown product of NO—is measured. In brief, 100 μl of cell culture supernatant was used after 72 h of *in vitro* cultivation. The Griess assay was performed as described before (Meisel et al., 2011). The nitrite content was calculated by extrapolation from a sodium nitrite standard curve assayed parallel to each measurement.

### Statistical Analysis

Results are indicated as means ± SD or ± SEM as indicated in figure legends. Statistical significances of differences in mean values were analyzed by using the unpaired two-tailed Student's *t*-test (GraphPad Prism). Significant differences were indicated with asterisks (^*^p ≤ 0.05; ^**^p ≤ 0.001; ^***^p ≤ 0.0001).

## Results

### Expression of mIDO1 mRNA and Protein in Lungs of Infected Mice

**Figure 1:**
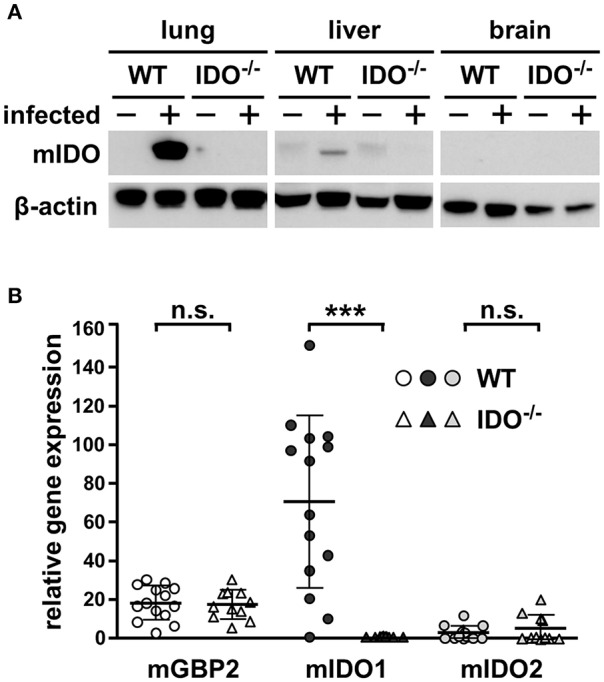
Expression of mIDO1 in murine lung tissue during *Toxoplasma gondii* infection. Gene and protein expression in tissues from naïve or *T. gondii* ME49-infected wild-type (WT) and indoleamine 2,3-dioxygenase 1-deficient (IDO^−/−^) mice. Western blot analysis shows murine IDO (mIDO) and β-actin protein in lung, liver, and brain tissue of naïve (–) and infected (+) WT and IDO^−/−^ mice **(A)**. Expression of murine guanylate binding protein 2 (mGBP2), mIDO1, and mIDO2 in lung tissue homogenates of infected mice relative to their expression in naïve control samples **(B)**. Data were normalized to the housekeeping gene β-actin and were represented as 2^−ΔΔ*CT*^ (naïve vs. infected) in scattered dot plots and means ± standard deviation. The Student's *t*-test (unpaired, two-tailed) was used to determine statistical differences marked with asterisks (n.s., not significant; ***p ≤ 0.0001).

To clear up potentially different murine indoleamine 2,3-dioxygenase 1 (mIDO1) distribution among various murine tissues within WT and IDO^−/−^ mice, Western blot analyses were performed. Here, no mIDO protein was detectable in liver, brain, or lung tissue of naïve WT mice, while infection induced strong mIDO1 expression in lung tissue as well as a slight expression in liver tissue. As suggested in IDO^−/−^ mice, in all tested conditions and tissues, no mIDO protein was detectable (Figure 1A). Further quantitative real-time PCR experiments with the lung tissues were conducted to detect mIDO1 mRNA. Shown data represent the relative gene expression of infected to naïve mice in WT and IDO^−/−^, respectively. Expression of mGBP2 was equally strong in WT and IDO^−/−^ mice 7 dpi, as expected. Upon *T. gondii* infection, mIDO1 mRNA expression is strongly upregulated in WT mice. As expected, we did not detect any mIDO1 expression in infected IDO^−/−^ mice. Murine mIDO2 was measured as well to exclude mIDO2 as a responsible candidate for results shown further on. mIDO2 is only marginally increased during *T. gondii* infection in a few infected mice and there is no significant difference between WT and IDO^−/−^ mice (Figure 1B). Additionally, we measured miNOS expression in lungs of WT and IDO^−/−^ mice at different time points post-infection *via* quantitative real-time PCR and Western blot analysis (Supplementary Figure S1). Relative miNOS expression in infected WT mice was increased in a time-dependent manner (Supplementary Figure S1A). However, differences between WT and IDO^−/−^ on day 7 post-infection were not significant (Supplementary Figure S1B). miNOS protein was absent in lungs of naïve WT and IDO^−/−^ mice. Infected IDO^−/−^ mice were positive for miNOS protein at 7 and 9 dpi and infected WT mice only at 9 dpi (Supplementary Figure S1C).

### Comparison of mIDO1-Based Tryptophan Degradation and Parasite Loads of Naïve and Infected WT and IDO^−/−^ Animals

We explored the antiparasitic properties influenced by mIDO1 during *T. gondii* infection by comparing the tryptophan degradation as well as the parasite load.

Therefore, we determined tryptophan and kynurenine concentrations in sera *via* HPLC analyses to analyze the systemic distribution of these metabolites. Furthermore, we analyzed lung tissue *via* HPLC, since we previously identified lung tissue as one center of mIDO1 protein and mRNA expression.

**Figure 2:**
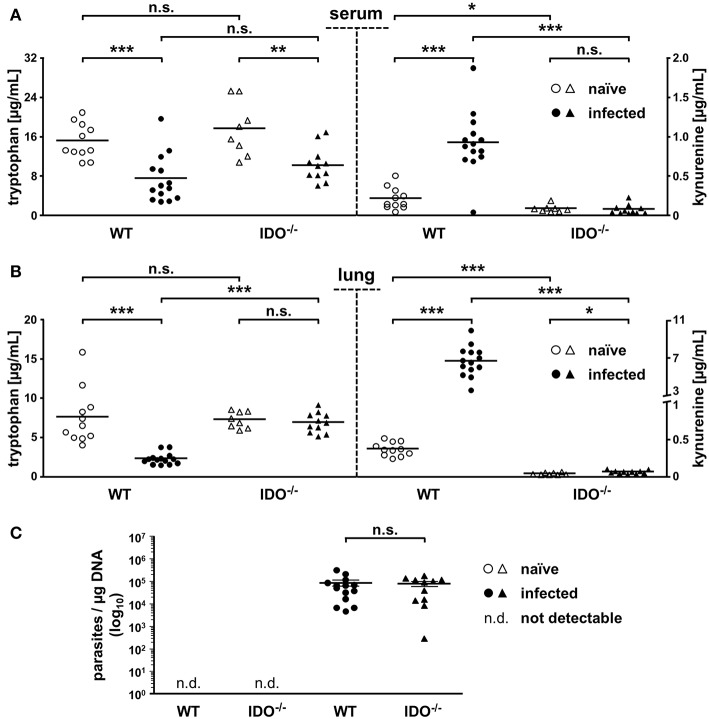
mIDO1 activity in murine lung tissue during *Toxoplasma gondii* infection. Serum and lung samples were collected from naïve or *T. gondii* ME49-infected wild-type (WT) and indoleamine 2,3-dioxygenase 1-deficient (IDO^−/−^) mice. Free tryptophan and kynurenine was measured *via* high-performance liquid chromatography in serum **(A)** and lung tissue homogenate samples **(B)**. Comparison of the *T. gondii* load (determined by B1-gene detection) in lungs of naïve or infected WT and IDO^−/−^ mice **(C)**. Data are represented as scattered dot plots and means (±standard error of the mean for RT-PCR data). For the RT-PCR data, each dot represents the mean of duplicate measurements of a single mouse lung tissue sample. The Student's *t*-test (unpaired, two-tailed) was used to determine statistical differences marked with asterisks (n.s., not significant; *p ≤ 0.05, **p ≤ 0.001, and ***p ≤ 0.0001).

With 15.3 and 17.7 μg/ml, naïve WT and IDO^−/−^ mice exhibit no significant differences in mean tryptophan concentrations in serum. On day 7 after *T. gondii* infection, serum tryptophan concentrations drop significantly to 7.5 and 10.2 μg/ml in WT and IDO^−/−^ animals, respectively. Concomitantly with this serum tryptophan drop, the serum kynurenine concentration in the WT rises significantly from 0.23 to 0.94 μg/ml. Even though the serum tryptophan concentration drops in the IDO^−/−^ animals similar to the WT, the serum kynurenine concentration is unaltered (<0.1 μg/ml) in the IDO^−/−^ animals (Figure 2A). The lung tryptophan concentrations of naïve WT and IDO^−/−^ mice behaved like the serum tryptophan concentrations without significant differences, but with 7.7 and 7.3 μg/ml, they are overall lower. Infected WT animals show a significant drop in lung tryptophan concentration (from 7.7 to 2.4 μg/ml) paired with a significant increase in the lung kynurenine concentration (from 0.4 to 6.7 μg/ml). In contrast, infected IDO^−/−^ animals exhibit no significant difference in lung tryptophan concentrations compared with the naïve group. Kynurenine concentrations in lungs of naïve and infected IDO^−/−^ animals are likewise low (<0.1 μg/ml) as in sera (Figure 2B).

To draw a conclusion regarding the previously mentioned potential antiparasitic properties of mIDO1, we determined the parasite load in lung tissues *via* real-time PCR from the same samples we analyzed beforehand. Here, we used specific oligonucleotides to detect the 35-fold repetitive B1 gene of *T. gondii*. As expected, there was no detection of *T. gondii* in naïve WT and IDO^−/−^ mice. We measured a significant amount of parasites in the lungs of WT and IDO^−/−^ mice 7 dpi; however, the parasite load in WT and IDO^−/−^ mice was not significantly different (Figure 2C).

### Suppressed T Cell Proliferation Responses During Acute *T. gondii* Infection

**Figure 3:**
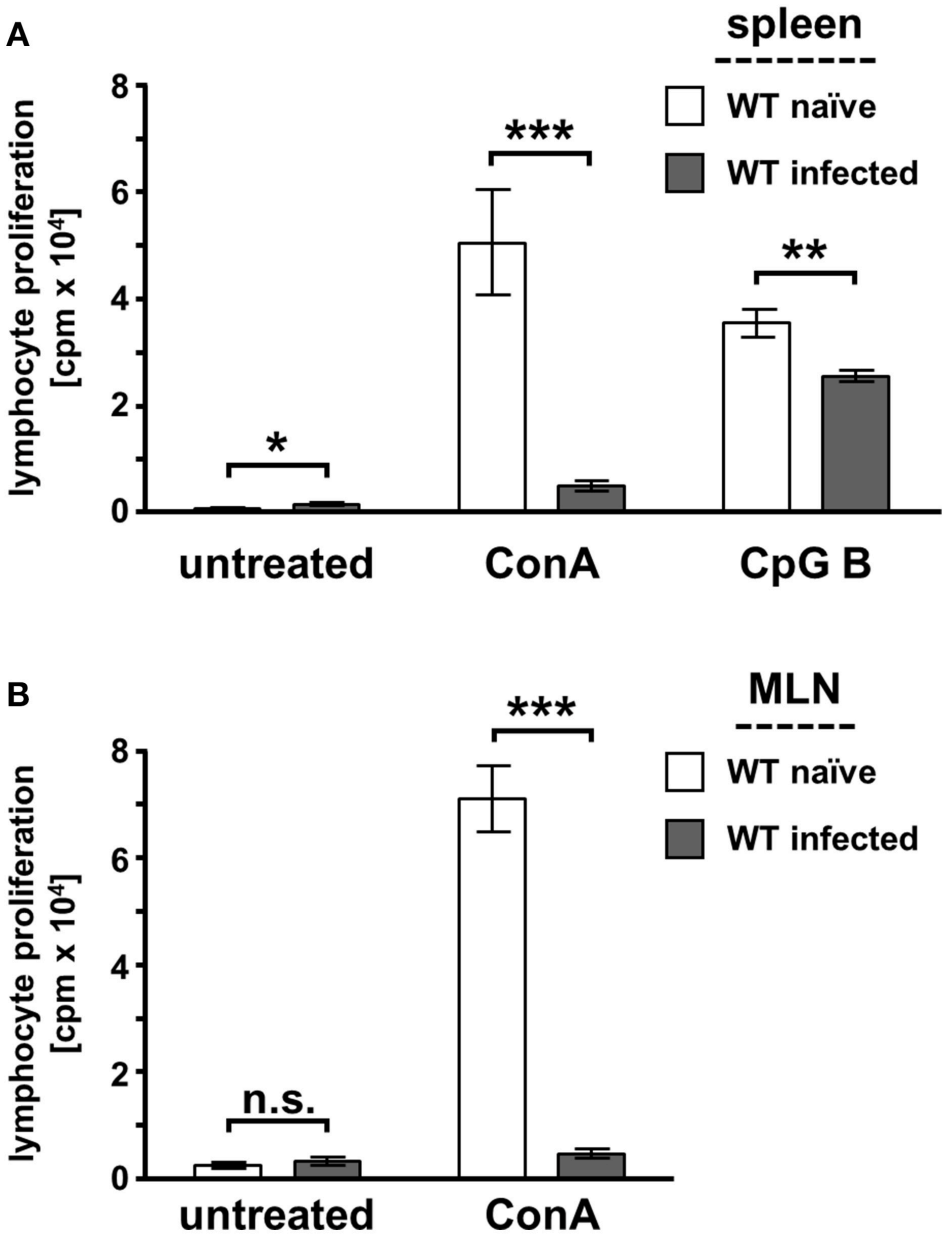
Mitogen-stimulated lymphocyte proliferation is impaired during acute toxoplasmosis in WT spleen and MLN-derived cells. Spleen **(A)** and mesenteric lymph nodes (MLN) **(B)** were isolated from naïve or *T. gondii* ME49-infected wild-type (WT) mice. Cell cultures were supplemented with the mitogen concanavalin A (ConA; 1 μg/ml), the class B phosphate linked cytosine, and guanine oligonucleotide ODN1826 (CpG B; 0.1 μM) to stimulate T cell and B cell proliferation *ex vivo* or were left untreated. Lymphocyte proliferation was determined with the ^3^H-thymidine method. Data were represented as means of triplicate measurements (*n* = 7–9) ± standard error of the mean. The Student's *t*-test (unpaired, two-tailed) was used to determine statistical differences marked with asterisks (n.s., not significant; *p ≤ 0.05, **p ≤ 0.001 and ***p ≤ 0.0001).

Splenocytes were isolated from naïve and infected WT mice to analyze the proliferative responses of lymphocytes during acute toxoplasmosis. We performed initial T cell proliferation experiments to analyze the suitability of our specifically adjusted experimental model (Supplementary Figure S2). Therefore, we infected WT mice i.p. with *in vitro* cultivated tachyzoites or bradyzoites isolated from lysed brain cysts propagated *in vivo*. Here, we could not detect any differences in mitogen-stimulated T cell proliferation responses (Supplementary Figure S2A). Furthermore, we tested the time-dependent mitogen-stimulated T cell proliferation responses. The results shown in Supplementary Figure S2B clearly illustrate that the proliferation responses are not impaired at 3 dpi, are reversibly impaired at 7 dpi, and are irreversibly impaired at 10 dpi (Supplementary Figure S2). The proliferation of T cells and B cells was induced by stimulation with the mitogens concanavalin A (ConA) and the class B CpG oligonucleotide ODN1826, respectively (Figure 3A). Untreated splenocytes from infected WT mice show a weak basal proliferation compared to splenocytes from naïve mice. Mitogen stimulation of naïve splenocytes induced a potent lymphocyte proliferation response, whereas splenocytes from infected mice have a low proliferation response. In more detail, CpG B stimulation showed that the B cell proliferation response was slightly reduced by approximately 28% during acute *T. gondii* infection. However, T cell stimulation with ConA showed a very prominent impaired T cell proliferation response (>90% reduction). ConA stimulation of MLN cells from naïve and infected WT mice resulted in the same phenotype (Figure 3B). In detail, MLN-derived T cells also showed a very weak proliferative response to *ex vivo* ConA stimulation during acute *T. gondii* infection (>90% reduction) as observed in the stimulation of splenic T cells. Thus, further experiments were conducted with splenocytes to perform more profound analyses of the T cell responses during acute toxoplasmosis.

### IL-2 Availability and NOS Activity, but Not mIDO1 Influence T Cell Proliferation Responses During Acute Toxoplasmosis

The role of mIDO1 in the suppressed T cell proliferation responses during acute toxoplasmosis is not known. Thus, splenocytes were isolated from naïve and *T. gondii*-infected WT and IDO^−/−^ mice 7 dpi.

**Figure 4:**
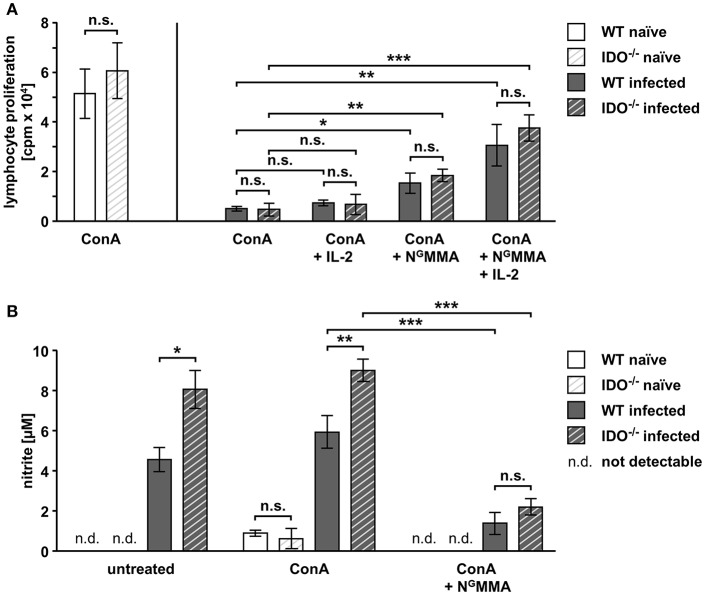
T cell proliferation is altered by IL-2 and NOS activity in splenocytes derived from WT and IDO^−/−^ mice. Splenocytes were isolated from naïve or *T. gondii* ME49-infected wild-type (WT) and indoleamine 2,3-dioxygenase 1-deficient (IDO^−/−^) mice (WT naïve: *n* = 7; IDO^−/−^ naïve: *n* = 5; WT infected: *n* = 8; IDO^−/−^ infected: *n* = 10). Splenic T cell cultures were stimulated with the mitogen concanavalin A (ConA, 1 μg/ml) *ex vivo*. Supplementation of human interleukin-2 (IL-2, 5 ng/ml) and the nitric oxide synthase (NOS) inhibitor N^G^-monomethyl-L-arginine (N^G^MMA, 100 μg/ml) was done as indicated **(A,B)**. Lymphocyte proliferation was determined with the ^3^H-thymidine method. **(A)**. Nitrite accumulation in the supernatant of *ex vivo* cultured splenocytes was detected using the Griess reaction **(B)**. Data were represented as means of triplicate measurements ± standard error of the mean. The Student's *t*-test (unpaired, two-tailed) was used to determine statistical differences marked with asterisks (n.s. = not significant; *p ≤ 0.05, **p ≤ 0.001, and ***p ≤ 0.0001).

Splenic T cells from naïve WT and IDO^−/−^ mice respond comparably strong to mitogen stimulation (Figure 4A). During acute *T. gondii* infection, the mitogen-induced proliferative responses were highly reduced in WT splenocytes (>90%) and IDO^−/−^ splenocytes (>92%) (Figure 4A). This indicates that the suppressed T cell responses are affected independently of mIDO1.

IL-2 deprivation (Khan et al., 1996) as well as iNOS activity (Patton et al., 2002) have previously been described to be involved in the impaired T cell proliferation response during acute toxoplasmosis. Thus, we performed *ex vivo* mitogen stimulation experiments with supplementation of IL-2 and the NOS inhibitor N^G^MMA to elucidate their interplay in the proliferation of splenocytes from WT and IDO^−/−^ mice. Supplementation of IL-2 alone did not significantly improve the proliferation of T cells from either infected genotype (WT: from 9.7 to 14.1%; IDO^−/−^: from 7.6 to 10.9%) (Figure 4A). NOS inhibition *via ex vivo* N^G^MMA treatment resulted in a small but significant elevation of T cell proliferation, which was equally strong for cells from both infected genotypes (WT: from 9.7 to 29.7%; IDO^−/−^: from 7.6 to 30.2%) (Figure 4A). Combining the supplementation of IL-2 and N^G^MMA increased the proliferation of mitogen-treated T cells from both infected genotypes even further, without however reaching the level of the naïve proliferation response (WT: from 9.7 to 59.5%; IDO^−/−^: from 7.6 to 61.8%) (Figure 4A), thus resulting in a highly significant elevation of the proliferative response compared to mitogen stimulation alone.

Stimulated as well as untreated splenocytes isolated from *T gondii*-infected WT and IDO^−/−^ mice showed NOS activity as measured indirectly *via* nitrite accumulation in the supernatant (Figure 4B). Here, splenocytes from IDO^−/−^ mice produce significantly more nitrite compared to the equally treated WT splenocytes (untreated: 4.6 μM for WT and 8.1 μM for IDO^−/−^; ConA stimulated: 5.9 μM for WT and 9 μM for IDO^−/−^) (Figure 4B). NOS inhibition *via ex vivo* N^G^MMA treatment reduced the nitrite concentration in supernatants strongly to 1.4 and 2.2 μM for WT and IDO^−/−^, respectively (Figure 4B).

## Discussion

Indoleamine 2,3-dioxygenase (IDO) is described as a potent antimicrobial factor in *in vitro* systems using human, porcine, and bovine cells. In this context, IDO activity has been shown to inhibit pathogens like bacteria (e.g., group A streptococci, *Staphylococcus aureus*), viruses (e.g., Herpes simplex virus 1, Cytomegalovirus), and parasites (e.g., *T. gondii, Neospora caninum*) (Däubener et al., 2009). However, the role of IDO as a potent antimicrobial factor *in vivo* remains controversial. Here, we used C57BL/6 mice deficient for mIDO1 (IDO^−/−^) to investigate acute toxoplasmosis with specific regard to the general systemic proinflammatory reaction and the local parasite burden within the lung, a strong IDO-expressing organ. We have adjusted our experimental model by comparing the infection with tachyzoites and bradyzoites. Furthermore, we have evaluated the optimal time point for our objectives. In this specifically adjusted experimental model, using a high dose of tachyzoites *via* the intraperitoneal route, C57BL/6 mice develop a more intense acute toxoplasmosis compared to BALB/c mice (own preliminary data not shown). The type II strain *T. gondii* ME49 was chosen for our infection experiments, since type II strains are the most frequently found *T. gondii* strains in human toxoplasmosis (Schlüter et al., 2014). To ensure standardized infection inoculums, we infected mice with a high dose of *T. gondii* ME49 tachyzoites *via* i.p. injection, thereby circumventing the use of brain-derived cysts that vary in size and parasite number (Dubey and Frenkel, 1998). However, it has to be taken into account that oral cyst uptake is a natural route of infection, whereas i.p. injection of tachyzoites into inbred mice is a strictly experimental setup. Additionally, infection *via* the natural route results in a slower but more natural course of disease compared to our specifically adjusted experimental model. Finally, it has to be considered that our model does not represent the natural course of toxoplasmosis, but is ideally adjusted for the herein analyzed objectives. Furthermore, it cannot be excluded that the high infection dose used in our model might mask a role of IDO during the natural course of a *T. gondii* infection.

Here, we show that infection of mice with *T. gondii* tachyzoites results in a strong mIDO1 induction in lungs. In detail, we found high amounts of mIDO1 mRNA and mIDO protein in the lungs of *T. gondii*-infected WT animals. Similar observations have been obtained during allergic diseases (Hayashi et al., 2004) or allogeneic stem cell transplantation (Lee et al., 2017) in mice. In both publications, mIDO immunoreactivity was found especially in lung epithelial cells (Hayashi et al., 2004; Lee et al., 2017). Furthermore, published data in the context of other murine infections have shown similar mIDO expression. mIDO protein and/or mRNA was found in the lungs of mice experimentally infected with the influenza A virus (Gaelings et al., 2017), the pathogenic fungus *Paracoccidioides brasiliensis* (Araújo et al., 2014), and the pathogenic bacterium *Mycobacterium tuberculosis* (*M. tuberculosis*) (Blumenthal et al., 2012), thus indicating that mIDO does function as an antimicrobial effector mechanism in murine lungs *in vivo*.

During *T. gondii* infection, we found reduced tryptophan concentrations in sera of WT animals, which were even more pronounced in lung tissue, confirming previously published data (Silva et al., 2002; Murakami et al., 2012). The same samples were tested for their kynurenine concentration, as kynurenine is a degradation product of tryptophan. The decreased tryptophan concentrations were accompanied with an increase in kynurenine concentrations. However, we also detected a drop in tryptophan concentrations in infected IDO^−/−^ mice. This observation is unlikely due to possible mIDO2 activity, since we did not detect mIDO2 mRNA in the majority of samples. Furthermore, the tryptophan drop in the IDO^−/−^ mice is not accompanied by an increase of kynurenine. Therefore, we suggest that enhanced protein biosynthesis by host cells and the rapidly proliferating *T. gondii* tachyzoites during the acute phase of toxoplasmosis are responsible for the decreased tryptophan concentration in the serum of infected animals. Our finding supports this hypothesis, since there is no evidence for an enhanced tryptophan cleavage in the lungs of infected, IDO^−/−^ mice.

We can confirm the observation by Divanovic et al. that IDO^−/−^ mice show no phenotype compared to the WT during acute toxoplasmosis, but rather behave similarly (data not shown). Furthermore, they reported that treatment of chronically infected WT mice with the IDO inhibitor 1-methyl-D-tryptophan (1-D-MT) resulted in *T. gondii* encephalitis (Divanovic et al., 2012). In a previous publication, Murakami et al. reported reduced mRNA expression of the *T. gondii* surface antigen 2 in lungs of *T. gondii*-infected IDO^−/−^ mice compared to WT mice 7 dpi, indicating a lower parasite load or a reduced metabolic activity. Therefore, we analyzed the parasite load in the lungs of the infected animals by detecting a *T. gondii*-specific DNA sequence. Here, we did not detect a significant difference in the *T. gondii* load in lungs of WT or IDO^−/−^ mice. Again, a possible involvement of mIDO2 to compensate for the lack of mIDO1 is unlikely, since mIDO2 mRNA was rare and detectable only at low levels in infected IDO^−/−^ and WT mice. Another tryptophan-degrading enzyme—tryptophan 2,3-dioxygenase (TDO)—might, however, be involved. Human TDO has been described by us to mediate antimicrobial and immunoregulatory effects similar to human IDO (Schmidt et al., 2009). Human TDO has been identified by Hsu et al. (2016) as the main tryptophan-degrading enzyme in human lung cancer-associated fibroblasts. Due to these findings, we have recently established a mIDO1 and mTDO double-deficient mouse strain to further elucidate the involvement of mIDO1 and mTDO during murine infections.

We have shown that a tryptophan concentration of <1 μg/ml is necessary to inhibit bacterial (*S. aureus*) growth as well as human T cell proliferation *in vitro* (Müller et al., 2009). Despite our current finding that the tryptophan concentration in murine lung tissue is strongly reduced during *T. gondii* infection, we could not detect increased parasite loads in lungs of IDO^−/−^ animals, even though *T. gondii* is tryptophan auxotroph. Thus, the tryptophan depletion 7 dpi might not be sufficient to mediate antiparasitic effects *in vivo*. Detailed information concerning the minimal tryptophan concentration for *T. gondii* growth *in vivo* is not available. Our data clearly showed a time-dependent increase of miNOS in lung tissue of infected WT mice on transcript level but could not detect a difference between WT and IDO^−/−^ mice 7 dpi. However, in lungs of IDO^−/−^ mice, we could detect miNOS protein earlier post-infection compared to the WT. Thus, iNOS expression in murine tissues might mediate parasite control during acute toxoplasmosis. This might be another reason why we did not find mIDO1 to be involved in the control of the rapidly replicating tachyzoites during acute toxoplasmosis. iNOS is a previously described antimicrobial defense mechanism and is induced in *T. gondii*-infected mice (Khan et al., 1997). However, mice deficient for iNOS showed prolonged survival in comparison to WT mice (Khan et al., 1997). Detailed analyses showed that enhanced liver degeneration, extensive ulceration, and necrosis in the small intestine were responsible for the earlier death of iNOS-expressing WT mice (Khan et al., 1997). We found higher nitrite accumulation in supernatants of *ex vivo* splenocyte cultures from infected IDO^−/−^ mice compared to infected WT mice. Ye et al. have reported a similar observation in a stem cell transplantation model. They showed that 1-methyl-DL-tryptophan mediated inhibition of mIDO resulted in increased NO concentration in the supernatant of mixed lymphocyte cultures with lymphocytes isolated from BALB/c and C57BL/6 mice (Ye et al., 2017). This indicates, on the one hand, that IDO is influencing NO production. On the other hand, we (Däubener et al., 1999) and others (Bando et al., 2018) found that iNOS can block IDO-mediated antimicrobial effects. Thus, we suggest that mIDO1 and iNOS interact during acute toxoplasmosis and that mIDO1 activity might be required for the regulation of iNOS activity during acute toxoplasmosis in WT mice. Higher iNOS activity might compensate for the missing mIDO1 in IDO^−/−^ mice, whereby potential antiparasitic effects of mIDO1 were not detectable in our experimental setup. The herein mentioned detection of miNOS protein supports this suggestion. Thus, infection experiments with mice deficient for mIDO1 and iNOS might be of interest, since Scharton-Kersten et al. (1997) reported that iNOS-deficient mice can survive acute toxoplasmosis and control parasite growth at the site of infection *via* NO-independent mechanisms. This observation might be due to other aforementioned defense mechanisms (e.g., GTPases) or due to IDO activity. However, that remains to be shown.

Experimental evidence that IDO mediates antimicrobial effects directly *via* tryptophan depletion in mice came from *in vivo* experiments with bacterial infections. For example, Peng and Monack published that genes in the tryptophan biosynthesis pathway are essential for the pathogenic bacterium *Francisella novicida* (*F. novicida*) to multiply in lungs of C57BL/6 mice (Peng and Monack, 2010). Thereafter, bacteria deficient in tryptophan synthesis were constructed, and it was found that this strain had lost its capacity to replicate in the lungs of C57BL/6 mice. In lungs of IDO^−/−^ mice, this tryptophan auxotrophic *F. novicida* strain was able to replicate, thus suggesting that tryptophan depletion *via* mIDO1 did protect the WT mice from the bacterial infection. Comparable data were obtained with a pharmacologic blockage of tryptophan synthesis in *M. tuberculosis*. Zhang et al. showed that a blockage of tryptophan synthesis by halogenated anthranilate analogs disrupted tryptophan biosynthesis in *M. tuberculosis*. Treatment of infected mice with this compound resulted in an inhibition of bacterial growth (Zhang et al., 2013). Inhibition of IDO in macaques during experimental infections with *M. tuberculosis* led to reduced bacterial burden, indicating a better control of the *M. tuberculosis* infection in treated animals (Gautam et al., 2018). However, Gautam et al. used 1-D-MT, which is not an IDO inhibitor but is rather described to inhibit IDO-mediated immunoregulatory functions (Metz et al., 2012). Therefore, the observed effects in macaques might be due to an enhanced immune reaction against *M. tuberculosis*.

In mice, mIDO expressing plasmacytoid DCs have been reported to suppress T cell responses in tumor-draining lymph nodes (Munn et al., 2004). Furthermore, DCs that express IDO have been linked to several other immunoregulatory functions, for example, the differentiation of regulatory T cells (Grohmann et al., 2017). In addition, tolerance toward self-antigens is regulated by mIDO in the marginal zones of the murine spleen (Ravishankar et al., 2012). Therefore, it was of interest to analyze whether mIDO1 is involved in the T cell suppression, seen during an acute *T. gondii*-infected mouse.

We measured T cell responses from *in vitro* mitogen-stimulated splenocytes, isolated from *T. gondii*-infected mice. Here, we observed a strong suppression of the T cell proliferation in splenocytes from infected compared to naïve mice. However, there was no difference between IDO^−/−^ and WT mice in our experiments, indicating that mIDO1 is not a major factor that regulates the observed T cell suppression. Previous experiments by Chan et al. (1986) have indicated that IL-2 availability as well as macrophages (as potential NO producers) are involved in the T cell suppression observed during acute toxoplasmosis.

Supplementation of IL-2 alone did not influence the proliferation of T cells in our setup, as reported by Khan and coworkers. They observed an increase in the T cell proliferation upon *in vitro* supplementation of IL-2 during mitogen stimulation of purified CD4^+^ T cells (Khan et al., 1996). In our setup, we stimulated splenocytes consisting not only of T cells but rather of a broad variety of cell types, including macrophages. T cell proliferation has also been reported to be influenced by NO derived from activated macrophage before (Albina et al., 1991; Patton et al., 2002). Inhibition of NOS in our experiments increased the proliferation of T cells derived from both infected mouse strains significantly. IL-2 supplementation and NOS inhibition in combination further increased T cell proliferation, but it did not reach the proliferation level of naïve T cells.

Salinas et al. (2014) have demonstrated that conventional T cells compete with regulatory T cells for available IL-2 in purified T cells isolated during acute toxoplasmosis, induced by infection of C57BL/6 mice orally with 50 *T. gondii* ME49 cysts. With our finding that splenocytes from IDO^−/−^ mice behave like splenocytes from WT mice during *ex vivo* mitogen stimulation, we suggest that the T cell suppression during acute toxoplasmosis is mediated by NOS activity and might even be mediated by IL-2 deprivation as described by Salinas et al. (2014). However,in that case, induction of regulatory T cells would then be independent of mIDO1 in the described T cell suppression during acute toxoplasmosis.

## Ethics Statement

This study was performed in strict compliance with the German Animal Welfare Act. The experiments were authorized by the North Rhine-Westphalia State Agency for Nature, Environment and Consumer Protection (Permit# 84 02.04.2013.A271, 84 02.04.2013.A495 and 84 02.04.2016.A508). All efforts were made to minimize animal suffering during the experiments.

## Author Contributions

WD and IF conceived and supervised the study. C-MU, AD, and WD designed the experiments, prepared the figures, and wrote the manuscript. C-MU and AD performed the majority of experiments and analyzed the data. TB, AT, SC, SE, and KS-B performed the experiments. US supervised animal experiments. All authors reviewed the manuscript.

### Conflict of Interest Statement

The authors declare that the research was conducted in the absence of any commercial or financial relationships that could be construed as a potential conflict of interest.

## Abbreviations

ConA: concanavalin A
CpG B: class B phosphate linked cytosine and guanine oligonucleotide
DC: dendritic cell
dpi: days post-infection
EDTA: ethylenediaminetetraacetic acid
FBS: fetal bovine serum
GBP: guanylate binding protein
GTPase: enzyme that hydrolyzes guanosine triphosphate
HFF: human foreskin fibroblasts
HPLC: high-performance liquid chromatography
i. p.: intraperitoneal
IDO: indoleamine 2,3-dioxygenase
IL-2: interleukin-2
IMDM: Isocove's Modified Dulbecco's Medium
inf: infected
IFN-γ: interferon gamma
iNOS: inducible nitric oxide synthase
IRG: immunity-related GTPase
MLN: mesenteric lymph node
n.d.: not detectable
n.s.: not significant
N^G^MMA: N^G^-monomethyl-L-arginine
NO: nitric oxide
PBS: phosphate-buffered saline
qRT PCR: quantitative real-time polymerase chain reaction
SD: standard deviation
SEM: standard error of the mean
TDO: tryptophan 2,3-dioxygenase
WT: wild type
1-L-MT: 1-methyl-L-tryptophan.
