# Supplementary material for: Indoleamine 2,3-Dioxygenase Activity During Acute Toxoplasmosis and the Suppressed T Cell Proliferation in Mice
Source: Front Cell Infect Microbiol. 2019 Jun 5;9:184. doi: 10.3389/fcimb.2019.00184 (PMC6561234; doi:10.3389/fcimb.2019.00184)
Supplement: Supplementary file 1 [file Data_Sheet_1.pdf]

Supplementary Material

Indoleamine 2,3-Dioxygenase Activity During Acute Toxoplasmosis and the Suppressed T Cell Proliferation in Mice

Christoph-Martin Ufermann[1†], Andreas Domröse[1†], Timo Babel[1], Anne Tersteegen[1], Sevgi Can Cengiz[2], Silvia Kathrin Eller[1], Katrin Spekker-Bosker[1], Ursula Regina Sorg[1], Irmgard Förster[2], Walter Däubener[1*]

[1] Institute of Medical Microbiology and Hospital Hygiene, Heinrich-Heine-University Düsseldorf, Düsseldorf, Germany
[2] Immunology and Environment, Life & Medical Sciences (LIMES) Institute, University of Bonn, Bonn, Germany.

[†]These authors have contributed equally to this work

***Correspondence:**

Prof. Dr. Walter Däubener
Institute of Medical Microbiology and Hospital Hygiene
Heinrich-Heine-University Düsseldorf
Universitätsstr. 1, Bldg. 22.21
40225 Düsseldorf, Germany
Phone: +49 (0) 211-81-12464
Fax: +49 (0) 211-81-15906
e-mail: daeubene@uni-duesseldorf.de

Content **Page**

Supplementary Table S1 Oligonucleotides used in this study **S-2**
Supplementary Figure S1 Expression of miNOS in murine lung tissue during *Toxoplasma gondii* infection **S-3**
Supplementary Figure S2 Mitogen induced lymphocytes proliferation responses in murine splenocytes **S-5**

Supplementary Table S1 Oligonucleotides used in this study.

Gene expression		
**Primer Name**	**Primer Sequence [5’->3’]**	**Roche Probe ID**
mβ-actin-fw	TGACAGGATGCAGAAGGAGA	106
mβ-actin-rv	CGCTCAGGAGGAGCAATG	
mGBP2-fw	TGAGTACCTGGAACATTCACTGAC	17
mGBP2-rv	AGTCGCGGCTCATTAAAGC	
mIDO1-fw	GGGCTTCTTCCTCGTCTCTC	2
mIDO1-rv	TGGATACAGTGGGGATTGCT	
mIDO2-fw	GTCCTTGGGGAGATACCACA	12
mIDO2-rv	CCAAGGCTTGTAATGATCTGG	
miNOS-fw	CTTTGCCACGGACGAGAC	13
miNOS-rv	TGTACTCTGAGGGCTGACACA	
**Parasite load**		
**Primer Name**	**Primer Sequence [5’->3’]**	
*Tg*B1-fw	GCTAAAGGCGTCATTGCTGTT	
*Tg*B1-rv	GGCGGAACCAACGGAAAT	
*Tg*B1-probe	FAM-ATCGCAACGGAGTTCTTCCCAGACGT-BHQ1	

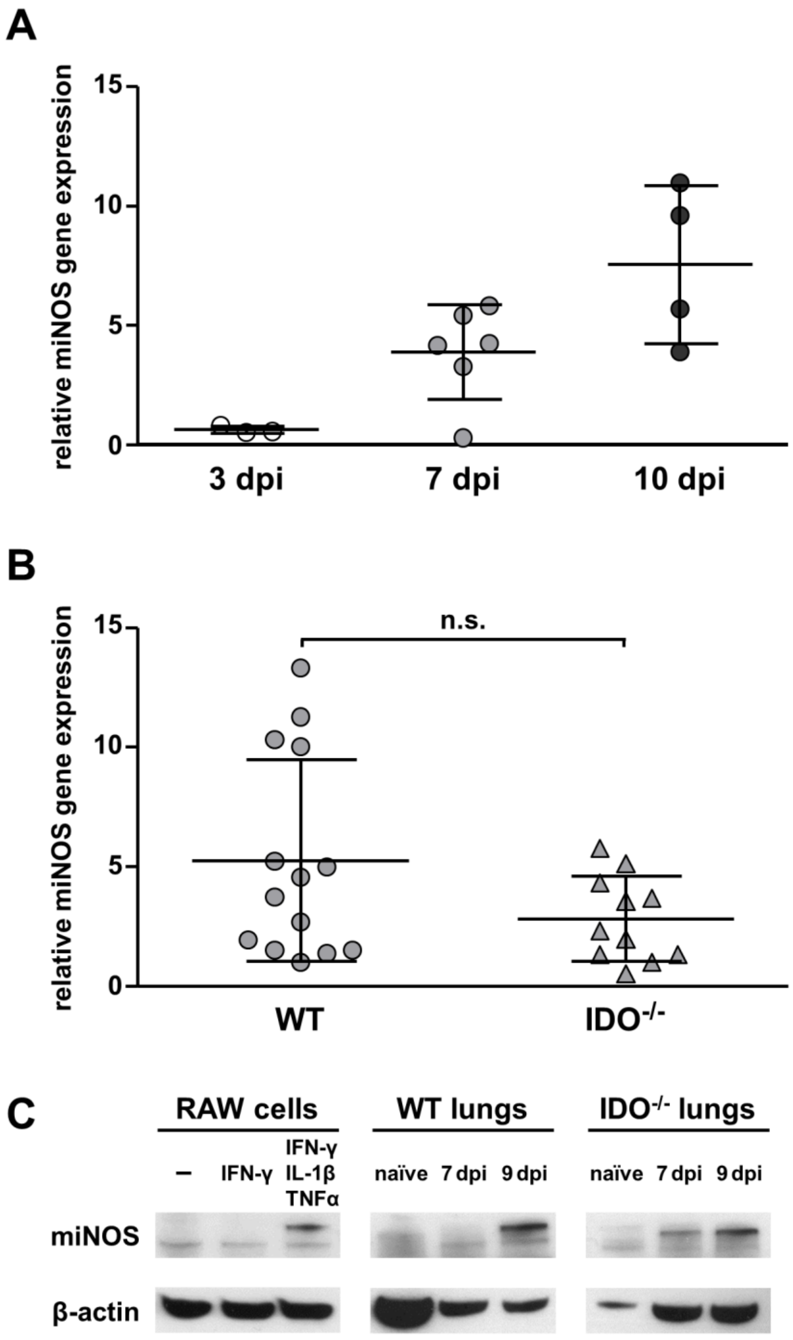

Supplementary Figure S1 Expression of miNOS in murine lung tissue during *Toxoplasma gondii* infection. Gene and protein expression of murine inducible nitric oxide synthase (miNOS) in tissues from naïve or *T. gondii* ME49 infected wild-type (WT) and indoleamine 2,3-dioxygenase 1-deficient ($IDO^{-/-}$) mice at different time points post infection. Expression of miNOS in lung tissue homogenates of infected mice relative to their expression in naïve control samples on 3, 7 and 10 days post infection (dpi) **(A)**. Expression of miNOS in lung tissue homogenates of infected WT and $IDO^{-/-}$ mice relative to their expression in naïve control samples at day 7 post infection (dpi) **(B)**. Western blot analysis shows miNOS and β-actin protein in lung tissue of naïve and infected WT and $IDO^{-/-}$ mice as well as untreated (-) and stimulated (IFN-γ 100 U/mL or IFN-γ 100 U/mL + IL-1β 100 U/mL + TNFα 100 U/mL) RAW 264.7 cells as negative and positive controls respectively **(C).** IFN-γ, IL-1β, TNFα

were purchased from R&D Systems (Minneapolis, USA). qPCR data were normalized to the housekeeping gene β-actin and were represented as $2^{-\Delta\Delta CT}$ (naïve vs. infected) in scattered dot plots and means ± standard deviation. The Student´s *t*-test (unpaired, two-tailed) was used to determine statistical differences marked with asterisks (n.s. = not significant).

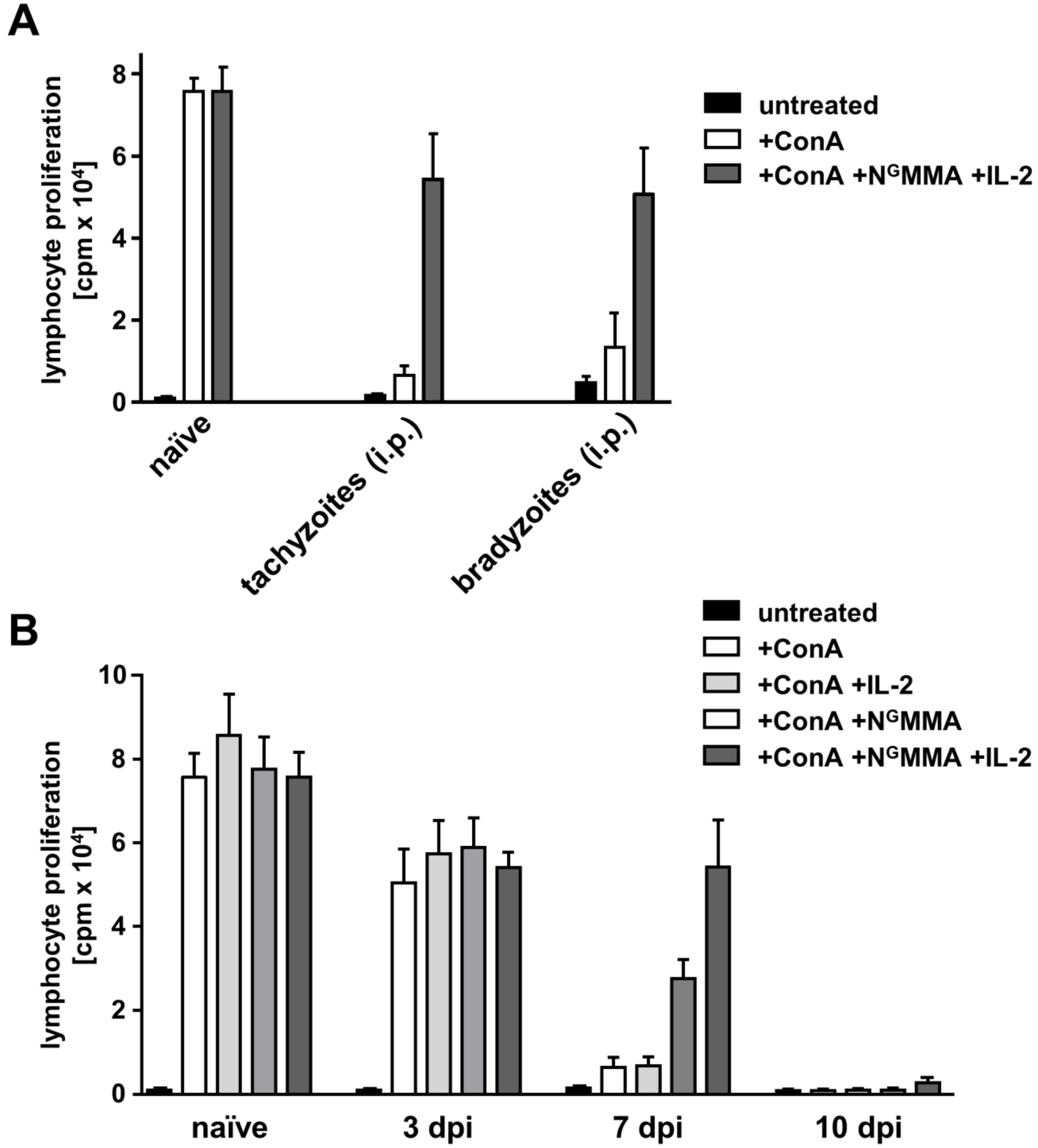

Supplementary Figure S2 Mitogen induced lymphocytes proliferation responses in murine splenocytes. Splenocytes were isolated from uninfected (naïve; n = 3) or *T. gondii* ME49 tachyzoite (intraperitoneal (i.p.) dose: 10^5 tachyzoites; n = 3) or bradyzoite (i.p. dose: 20 lysed cysts; n = 3) infected C57BL/6 wild-type (WT) mice at 7 days post infection (dpi) **(A)**. Splenocytes were isolated from uninfected (naïve; n = 3) or *T. gondii* ME49 tachyzoite infected (i.p. dose: 10^5 tachyzoites) WT mice at 3 dpi (n = 3), 7 dpi (n = 3) and 10 dpi (n = 3) **(B)**. Splenic T cell cultures were stimulated with the mitogen concanavalin A (ConA, 1 µg/mL) *ex vivo*. Additional supplementation of recombinant interleukin-2 (IL-2, 5 ng/mL) and the nitric oxide synthase (NOS) inhibitor

N^G-monomethyl-L-arginine (N^GMMA, 100 µg/mL) was done as indicated. Lymphocyte proliferation was determined with the ^{3}H-thymidine method. Data were represented as means of triplicate measurements + standard error of the mean of one experiment.
